# Decoding Movement From Electrocorticographic Activity: A Review

**DOI:** 10.3389/fninf.2019.00074

**Published:** 2019-12-03

**Authors:** Ksenia Volkova, Mikhail A. Lebedev, Alexander Kaplan, Alexei Ossadtchi

**Affiliations:** ^1^Center for Bioelectric Interfaces, Higher School of Economics, National Research University, Moscow, Russia; ^2^Center for Biotechnology Development, National Research Lobachevsky State University of Nizhny Novgorod, Nizhny Novgorod, Russia; ^3^Laboratory for Neurophysiology and Neuro-Computer Interfaces, Faculty of Biology, Lomonosov Moscow State University, Moscow, Russia

**Keywords:** electrocorticography, ECoG, brain-computer interface, BCI, movement decoding

## Abstract

Electrocorticography (ECoG) holds promise to provide efficient neuroprosthetic solutions for people suffering from neurological disabilities. This recording technique combines adequate temporal and spatial resolution with the lower risks of medical complications compared to the other invasive methods. ECoG is routinely used in clinical practice for preoperative cortical mapping in epileptic patients. During the last two decades, research utilizing ECoG has considerably grown, including the paradigms where behaviorally relevant information is extracted from ECoG activity with decoding algorithms of different complexity. Several research groups have advanced toward the development of assistive devices driven by brain-computer interfaces (BCIs) that decode motor commands from multichannel ECoG recordings. Here we review the evolution of this field and its recent tendencies, and discuss the potential areas for future development.

## 1. Introduction

The brain is a unique organ of the human body. Containing myriads of neurons, the brain circuits continuously process multiple sensory, motor and cognitive signals, generate thoughts and decisions, and produce a subjective feeling of being conscious and free-willed. The brain enables us with the capacity to effortlessly control such complex behaviors as voluntary movements of body parts, maintenance of posture and balance, speech production, and perception of the external world. Unfortunately, neurological disease or trauma may cause dramatic disruptions of these neuronal mechanisms, making an individual unable to move, feel and communicate. Many of such devastating neurological conditions currently have no cure, including amyotrophic lateral sclerosis (ALS), stroke, and spinal cord injury (SCI).

BCIs, also called brain-machine interfaces (BMIs) and neural prostheses, hold promise to provide revolutionary solutions to the treatment of brain disorders. BCIs connect neural circuits to external devices, such as prosthetic limbs, means of communication, computers, appliances for functional electrical stimulation, and even the other parts of the brain (Lebedev and Nicolelis, 2006). Medical applications of BCIs strive to restore functions lost to neurological disorders and aid in rehabilitation. For example, BCI approach to SCI consists of directly connecting the unaffected brain regions, such as the sensorimotor cortex, to a limb prosthesis (Hochberg et al., 2006, 2012; Collinger et al., 2013; Bouton et al., 2016). Many neuroprosthetic components have been proposed and developed over the last two decades. These are biocompatible implants for neural recordings, devices for stimulating neural circuits, and wireless recording systems. BCIs can connect the brain to computer cursors (Carmena et al., 2003; Lebedev et al., 2005), text generators (Pan et al., 2013; Akram et al., 2014), arm prostheses (Carmena et al., 2003; Velliste et al., 2008; Collinger et al., 2013), exoskeletons for assisted walking (Gancet et al., 2011; Contreras-Vidal and Grossman, 2013; Kwak et al., 2015), virtual-reality objects (Badia et al., 2013), powered wheelchairs (Galán et al., 2008; Chai et al., 2014), drones (LaFleur et al., 2013), and automobiles (Göhring et al., 2013). Recently, futuristic BCIs have emerged that merge several individual brains into a brain-net (Pais-Vieira et al., 2013; Rao et al., 2014).

Among different classes of BCIs, BCIs that operate in the motor domain have underwent a particularly extensive development because of the expectation that they could treat paralysis by enabling voluntary control of prosthetic limbs. Motor BCIs have been developed that enable movements of the arms (Wessberg et al., 2000; Carmena et al., 2003; Velliste et al., 2008; Collinger et al., 2013) and legs (Fitzsimmons et al., 2009). In addition to BCIs that enact movements, BCIs have emerged that handle cognitive functions, like executive control, attention, and decision making (Andersen et al., 2004, 2010; Mirabella and Lebedev, 2016). In the sensory domain, BCIs have been developed that apply stimulation to peripheral and central structures of the nervous system to evoke percepts mimicking natural senses, including hearing (House, 1976), vision (Dobelle, 2000; Normann et al., 2009), and touch (Bensmaia and Miller, 2014).

In this review, we focus on BCIs that are based on an invasive recording method called ECoG. We argue that ECoG could provide efficient solutions for many clinical cases because, first, ECoG grids sample neural signals with better spatial and temporal resolution compared to non-invasive recording methods, such as electroencephalography (EEG), and second, ECoG electrodes do not penetrate into the brain and thus offer a safer solution compared to the techniques that require insertion of recording sensors into the nervous tissue (Leuthardt et al., 2004; Hill et al., 2012; Petroff et al., 2016). The studies conducted up to date have demonstrated that ECoG-based BCIs are applicable to motor tasks. Yet, we suggest that accuracy of such motor BCIs could be improved by the implementation of more advanced neural decoding algorithms, particularly the ones based on deep neural networks.

We start with an overview of ECoG recording methods. Next, we review the motor tasks that have been utilized in ECoG decoding studies. Finally, we discuss the relevant decoding algorithms and software.

## 2. ECoG Methodology and Its Advantages Compared to the Other Recording Methods

A multitude of methods for recording of brain activity have been developed during the last several decades. Depending on the biological and physical principles employed, these methods have different spatial and temporal resolution. The recording methods range from classical single-unit techniques, where microelectrodes are inserted into the brain tissue, to non-invasive approaches, such as EEG, magnetoencephalography (MEG), near-infrared spectroscopy and functional magnetic resonance imaging. The choice of method in each concrete case is based on a number of requirements, including an assessment of risk to human subjects.

With the advancement of BCIs, we have seen a development of multichannel recording methods that allow sampling signals from many brain regions simultaneously (Nicolelis and Lebedev, 2009). To build clinically relevant neural prostheses, such recording methods should be viable for long periods of time. Chronically implanted multielectrode arrays (MEAs) measure brain activity at high spatial (at the level of single neurons) and temporal (at the level of neuronal spikes) resolution. MEAs-based BCIs have been implemented in rats (Chapin et al., 1999; Song et al., 2009), non-human primates (Taylor et al., 2002; Carmena et al., 2003; Gilja et al., 2012; Schaffelhofer et al., 2015) and humans (Hochberg et al., 2006; Collinger et al., 2013; Gilja et al., 2015; Brandman et al., 2017). The number of motor degrees of freedom that such BCIs could handle has been steadily growing (Hochberg et al., 2012; Collinger et al., 2013; Wodlinger et al., 2014; Vaskov et al., 2018). Recordings with MEAs are, however, not without problems, particularly when utilized in humans, since intracortical electrodes may provoke infection, tissue damage and scarring – the factors that contribute to deterioration of recording quality over time (Perge et al., 2013; Nuyujukian et al., 2014; Murphy et al., 2016; Kim et al., 2018).

While non-invasive BCIs do not have appreciable health risks, they have limitations of their own. Thus, EEG-based BCIs, which are currently prevalent because of their ease of use (Nicolas-Alonso and Gomez-Gil, 2012), have a lower information transfer rate compared to invasive BCIs (Lebedev and Nicolelis, 2006). Signal to noise ratio and spatial resolution are low for EEG recordings because with this method electrical potentials are sampled at a distance from their source, get smeared due to propagation through brain meninges and skull, and are susceptible to contamination with mechanical, electrooculographic (EOG), and electromyographic (EMG) artifacts (Cooper et al., 1965). Classification of several discrete motor states can be achieved with EEG recordings (for example, detecting the presence or absence of an actual or imagined limb movement). However, accurate decoding of fine movement characteristics is difficult with this method.

ECoG alleviates several problems related to using the other recording methods. With ECoG, electrical signal is recorded from the surface of the brain either epidurally (i.e., the electrodes are placed on the surface of the dura mater), or subdurally (i.e., the electrodes are placed underneath the dura mater.) While ECoG signals resemble EEG data (Kellis et al., 2016), they have greater amplitude, higher spatial resolution and broader frequency range (Schalk and Leuthardt, 2011). ECoG is superior to EEG for recordings of both cortical low-frequency oscillations (Hughes and Crunelli, 2005) and high-frequency activity in the gamma-range (Manning et al., 2009; Schalk and Leuthardt, 2011). The superior spatial and frequency resolution of ECoG enables obtaining detailed cortical maps, for example motor and sensory maps of individual fingers, while sampling electrical activity from many cortical areas simultaneously. Additionally, ECoG recordings are stable long-term (Blakely et al., 2009). By contrast, recordings of multiple single units with MEAs are not so stable, even though they could be considered a BCI control signal of superior quality. Although in the majority of studies ECoG grids have been implanted for a few days to minimize the infection risks associated to the use of tethered cables, it has been also shown that chronic ECoG implants are viable (Wyler et al., 1991; Weinand et al., 1994) and progress has been made toward the development of wireless, fully-implantable technologies (Vansteensel et al., 2016; Benabid et al., 2019). Based on these trends, it is reasonable to expect that clinically relevant, chronically implanted ECoG-based neural prostheses will emerge in the future for assisting patients suffering from neurological disorders. In summary, ECoG approach has multiple advantages for BCI applications, including an adequately high information transfer rate, stability of recordings, and a lower risk of medical complications. These features make ECoG method attractive for the developers of practical neuroprosthetic devices.

In clinical applications, ECoG electrodes are typically arranged into rectangular grids (for example, 6 × 8 or 8 × 8) or strips containing several electrodes in a single row. Platinum-iridium electrodes are often used, with the diameter of 4 mm most common for clinical applications. The commonly used 1-cm interelectrode distance yields an appropriate spatial resolution in many cases. Yet, the physical limit for resolution that could be achieved by decreasing the interelectrode distance is ~1.25 mm for subdural recordings (Freeman et al., 2000) and ~1.4 mm for epidural recordings (Slutzky et al., 2010). As a step toward reaching these limits of spatial resolution, ECoG grids with the spacing of 3–5 mm have been introduced and tested in a handful of studies conducted during the last decade (Wang et al., 2016). In such grids, neighboring electrodes carry sufficiently different information in the high gamma frequency range, as evident from the low coherence (~0.3) between their signals (Wang et al., 2009). These grids have a superior spatial resolution compared to the 1-cm spaced grids not only because of the narrower inter-electrode spacing but also because of the smaller electrode size, which aids sampling local activity. With the 3–5 mm electrode spacing, accurate classification of finger movements and multiple hand gestures has been achieved, as well as real-time control of a hand prosthesis (Wang et al., 2013; Bleichner et al., 2016; Hotson et al., 2016). More recently, even denser micro-ECoG grids have emerged with 40–80-micron wires and 1–3 mm spacing; these grid can occasionally sample activity of single cortical neurons (Khodagholy et al., 2015).

ECoG grids implanted for clinical reasons have been used as a testbed for different types of BCIs. With epidural ECoG recordings (a safer option for clinical assessment), BCIs have been implemented for reliably detecting movements (Chavarriaga et al., 2016), recognizing different movement types (Spüler et al., 2014b) and decoding movement time-course (Flint et al., 2016). For widely spaced ECoG electrodes, decoding accuracy with epidural grids is similar to that achieved with subdural electrodes (Spüler et al., 2014a). Yet, if high-density ECoG grids are used, they work better when implanted subdurally (Bundy et al., 2014). In theory, it is desirable to place ECoG implants over as many cortical sites as possible because motor planning and execution engage multiple cortical areas. However, using many implants increases the health risk. Several studies have attempted to optimize the number and placement of ECoG electrodes (Bleichner et al., 2016; Li et al., 2017). Intraoperative assessment of electrical activity at different cortical sites, before an ECoG grid is implanted (Xie et al., 2015), is one way to decrease the implant size and reduce the health risk.

## 3. Motor Paradigms

Movements can be decoded from the brain electrical activity owing to the existence of correlation between neural modulations and motor parameters, for a range of motor tasks (Lebedev, 2014). Thus, ECoG modulations are correlated with the movements of both the upper and lower limbs (Toro et al., 1994b; McCrimmon et al., 2017). BCI decoding algorithms convert neural modulations into the output signals of interest, such as limb position in space. While decoding algorithms are often evaluated offline using previously collected neuronal data, their ultimate testing should be conducted in real-time settings, where subjects control actions performed by an external device directly with their brain activity.

The development of new decoding algorithms not only advances BCIs by improving their accuracy of performance and versatility, but also leads to new fundamental insights regarding the brain motor, sensory and cognitive mechanisms, the insights that emerge during BCI experiments and their trouble shooting (Nicolelis and Lebedev, 2009). Specifically, research on ECoG-based BCIs provides insights on the encoding of movements and sensations by the collective activity of cortical neuronal populations, functional significance of different cortical rhythms, somatotopic representation of body parts, as evident from ECoG activity at different cortical sites and frequency bands, and the capacity of the brain to plastically adapt to novel BCI tasks.

A variety of movement types can be decoded from ECoG signals. These are wrist flexion and extension (Satow et al., 2003; Gharabaghi et al., 2014; Spüler et al., 2014a; Jiang et al., 2015, 2017), various grasp types (Graimann et al., 2003; Miller et al., 2007; Pistohl et al., 2012; Chestek et al., 2013; Xie et al., 2015) hand gestures and postures (Graimann et al., 2003; Chestek et al., 2013; Bleichner et al., 2016; Li et al., 2017), individual finger movement (Graimann et al., 2003; Kubanek et al., 2009; Miller et al., 2009; Samiee et al., 2010; Wang et al., 2011; Elghrabawy and Wahed, 2012; Flamary and Rakotomamonjy, 2012; Liang and Bougrain, 2012; Chestek et al., 2013; Chen et al., 2014; Xie et al., 2018), tongue and lip protrusion (Graimann et al., 2003; Satow et al., 2003; Miller et al., 2007; Paul et al., 2017), and foot movements (Toro et al., 1994b; Satow et al., 2003). While cortical areas contralateral to the moving body part are usually used for decoding, the option of using ipsilateral cortex has been considered as well (Hotson et al., 2014).

In real-time BCIs, signals representing movements or their imagery are decoded from ECoG activity and sent as control signals to external devices, such as screen cursor. Cursor control has been implemented in one (Leuthardt et al., 2004, 2006), two (Schalk et al., 2008), and three (Wang et al., 2013) dimensions. Additionally, ECoG-based BCIs have been demonstrated for the tasks of controlling a prosthetic hand (Yanagisawa et al., 2011; Chestek et al., 2013; Wang et al., 2013; Hotson et al., 2016; Li et al., 2017), enabling exoskeleton-assisted walking (Benabid et al., 2019), and selecting font characters with a speller application (Vansteensel et al., 2016).

**Figure 1:**
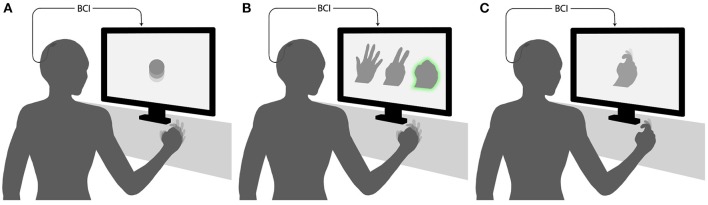
Experimental paradigms for decoding of movements from ECoG. **(A)** An arbitrary mapping paradigm, where the task performed by the subject and BCI output are dissimilar. In the illustrated example, clenching of the fist produces an upward movement of the pointer. **(B)** A discrete classification paradigm, where a BCI recognizes a posture or movement performed by the subject and reproduces it with an external device. The case is illustrated, where a subjects shapes his/her hand in one of three gestures, and the BCI generates a gesture of a virtual hand shown on the screen. **(C)** Continuous decoding paradigm, where movement parameters are decoded continuously (as a function of time or some other parameter) and reproduced by an external device. In the illustrated example, a virtual finger reproduces the trajectory of the subject's finger.

Here we focus on ECoG-based motor BCIs, which are BCIs where users modulate their cortical activity to generate movements of external devices. Such BCIs can be grouped into three main categories by the relationship between the task performed by the subject and BCI output (Figure 1) (while this classification can be applied to other types of BCIs, for example the ones based on EEG recordings, our review is restricted to ECoG-based systems). In the first category, there is an arbitrary relationship between the subject's action and the resulting movement of an external effector. For example, a subject imagines moving the hand to generate an upward movement of the pointer and imagines moving the tongue to move the pointer downward (Leuthardt et al., 2006). In the second category, a discrete classifier recognizes a motor action performed or imagined by the subject, for example moving one of the fingers. Next, an external device executes the same action. The third category of BCIs decode different motor parameters, such as movement direction, speed, acceleration, and force. The parameters are treated by the mathematical algorithm as continuous variables. An external device then reconstructs the movement from the decoded motor parameters.

### 3.1. Arbitrary-Mapping Paradigms

The arbitrary-mapping paradigm was the earliest to be implemented with ECoG recordings. The early studies employed event-related potentials for extracting motor commands (Toro et al., 1994b; Huggins et al., 1999; Levine et al., 1999). Later, ECoG spectral changes during real or imagined movements were used for BCI control (Leuthardt et al., 2004). In both groups of studies, actions performed by the subjects were mapped in an arbitrary way to actions executed by external devices.

To identify the most efficient control strategy for such an arbitrary-mapping BCI, Leuthardt et al. (2004) introduced a pre-screening procedure, which has become a common practice (Leuthardt et al., 2006; Miller et al., 2007; Schalk et al., 2008). During pre-screening, subjects perform a range of tasks so that ECoG features with the most prominent modulations could be identified and used for BCI control. The tasks are performed with the body parts represented by the cortical areas covered by the implanted ECoG electrodes (Schalk et al., 2008). Subjects perform or imagine motor acts like opening and closing the hand, protruding and retracting the tongue, flexing, and extending individual fingers, pursing and unpursing the lips, moving the arm, leg or foot (Miller et al., 2007), moving the jaw, shrugging the shoulders (Schalk et al., 2008), and pronouncing words (Leuthardt et al., 2004, 2006). Based on ECoG activity patterns exhibited during these tasks, subsets of ECoG features (e.g., frequency bands and electrodes with the most prominent modulations) are selected for implementing a BCI.

With the pre-screening approach, actions causing the largest ECoG modulations could be quickly selected to improve accuracy of BCI control. In a pioneering study (Leuthardt et al., 2004), subjects reached the success rates of 74–100% after 3–24 min of training in closed-loop experiments where they performed or imagined a preselected action (like opening and closing the hand, protruding the tongue or saying the word “move”) to move a screen cursor in the vertical dimension. In these experiments, ECoG grids were placed over frontal, parietal and temporal cortical areas. In the next study (Leuthardt et al., 2006), the same group added to the experimental design an adjustment procedure, where the decoder settings were updated using the data from the initial online session. This adjustment accounted for the differences between ECoG modulations exhibited during the pre-screening procedure and the online control.

Schalk et al. (2008) designed an arbitrary-mapping approach for the case of two-dimensional cursor movements. ECoG recordings were conducted from the frontal, temporal, and/or parietal cortex. During the pre-screening procedure, two tasks were selected that yielded the least correlated signal features (frequency bands and electrode locations) that were then used to independently control two coordinates of the cursor. After a training period of 12–26 min, five subjects achieved accuracy of 53–73% (with a 25% chance level) in a four-target task.

Wang et al. (2013) expanded the degrees of freedom of cursor movements to three dimensions. A tetraplegic subject with C4 level spinal cord injury underwent training for several weeks. ECoG activity was recorded using a high-density 32-electrode grid with 4-mm spacing; electrode diameter was 2 or 3 mm. The grid was implanted over the hand and arm representing areas of the left sensorimotor cortex. The subject learned to activate his sensorimotor cortex by attempting voluntary movements. Distinct cortical modulations occurred for attempted movements of different segments of the patient's upper limb. The BCI control consisted of assigning of each type of attempted movement to a particular direction of cursor movement. The decoder processed ECoG modulations in the gamma band. An adapting decoding scheme was used, where the decoder alternated between the periods when the decoder weights were fixed and when they underwent adjustments. The subject first learned a two-dimensional control of the cursor in a virtual environment, then the third dimension was added by gradually merging the weights calculated for the attempted three-dimensional task with the weights previously calculated for the two-dimensional control. The subject reached the success rate of 80% in the cursor control task, and also learned to control three-dimensional reaching movements performed by a prosthetic arm. In the next study conducted by the same group (Degenhart et al., 2018), two additional subjects with arm paralysis were tested, one with ALS and the other with brachial plexus injury. The subjects used a somatotopic control strategy to operate a virtual cursor in two or three dimensions. In this strategy, spatio-temporal patterns of gamma-band cortical activity evoked by different attempted upper-limb movements were converted into the direction of cursor movement. Cursor velocity was generated from ECoG gamma activity with an optimal linear estimator algorithm (Salinas and Abbott, 1994). Both subjects achieved control with up to three degrees of freedom.

Overall, the arbitrary-mapping approach has been shown suitable for building practical BCIs for the paralyzed patients capable of voluntarily modulating activity in the brain areas representing their paralyzed body parts (Spüler et al., 2014b; Chaudhary et al., 2016). Thus, Vansteensel et al. (2016) recently demonstrated a practical, a fully implanted ECoG-based BCI, where a patient with ALS learned to control a computer typing program by attempting voluntary hand movements. The ECoG grid was implanted subdurally over the motor cortex. This BCI enabled communication with a rate of two letters per minute. Notwithstanding the slow operation, BCIs of this kind offer a practical solution for functional restoration, communication and rehabilitation of neurologically impaired patients. As such, this approach needs to be further developed.

### 3.2. Classification and Reproduction of Movements

The second class of ECoG-based BCIs reproduces the same movements that subjects perform or imagine, which are recognized using a discrete classifier. High spatial and temporal resolution of ECoG allows recognizing a sufficiently large repertoire of movement types and executing them with an external device. Thus, areas corresponding to individual fingers can be discerned with ECoG recordings (Miller et al., 2009), which allows implementing a BCI that recognizes the finger being moved or imagined being moved with a classifier like Naïve Bayes (Chestek et al., 2013), linear discriminant analysis (LDA) (Wang et al., 2009; Hotson et al., 2016), or support vector machine (SVM) (Liu et al., 2010). Several studies have demonstrated that such classification can be performed with high accuracy based on ECoG recordings from the hemisphere contralateral to the working hand. Wang et al. (2009) decoded the finger being moved from the signals recorded with a micro-ECoG grid that was placed over the contralateral motor cortex. In this study, one subject performed self-paced finger flexions and extensions for ~10 s. The active finger was identified with an accuracy of 73% with both LDA that processed the ECoG data reduced to the first eight principal components and an SVM classifier without dimensionality reduction. In the study by Kubanek et al. (2009) subjects responded to a cue by flexing an individual finger 3–5 times over a time period of 1.5–3 s. ECoG activity was recorded from the frontal or temporal cortical areas. The relationship between the poser in different ECoG spectral bands and finger trajectories was modeled using a linear decoder called PaceRegression. The active finger was then determined as the finger with the highest decoded flexion amplitude. The across-subject average classification accuracy was 77.1% when ECoG activity recorded at movement onset was analyzed. The accuracy increased to 80.3% when the analysis interval was optimized for each subject. Hotson et al. (2016) applied a hierarchical LDA classification scheme to detect the finger being moved, reaching an accuracy of 76%. Furthermore, Liu et al. (2010) showed that ECoG activity in the sensorimotor cortex ipsilateral to the working hand could be used to determine the finger being moved. Their decoder incorporated logistic regression (LR) and a binary SVM.

Several studies have classified hand configuration from ECoG recordings. Yanagisawa et al. (2011) recorded ECoG activity in the sensorimotor cortex of a subject performing three types of hand movements: grasping, hand-opening, and scissor-mimicking movements. With these tasks, they implemented online control of a prosthetic hand based on a two-step classification scheme, where the first step consisted of detecting movement intention and the second step was the decoding of movement type. Linear SVM was used as classification algorithm for both steps. The intention to move was detected on average 37 ms earlier than the actual movement onset. Movement type was classified with the accuracy of 69.2%, which significantly exceeded the 33.3% chance level. Pistohl et al. (2008) employed regularized LDA to decode two types of grasping movements from the ECoG recorded over the motor cortex. They decoded the intention to move from ECoG 125-250 ms earlier than the actual movement onset. The subjects performed self-paced relocation of an object between several positions using either precision grip or whole-hand grasping. The grasp type was decoded with 93% accuracy based on the analysis of the time interval starting 1s before grasp till 0.5s after. Recording sites located anterior to the central sulcus were used for decoding whereas the sites posterior to the central sulcus were excluded as representing sensory responses.

Chestek et al. (2013) further increased the number of hand configurations decoded from the ECoG recorded over the sensorimotor-cortex. Their subjects configured the hands into one of four isometric postures: fist, pinch, point or five-finger spray. Additionally, the subjects flexed one or several fingers. The interval −0.5–1.5s relative to movement onset was used in this analysis. Classification was performed with a Naïve Bayes decoder applied to the gamma band of the ECoG. Four hand postures were classified with an accuracy of 68–81%, and 66–98% accuracy was achieved in a five-class classification, where classes represented four finger movements and a resting state. The same decoding methods were then utilized in the online sessions where subjects controlled a hand prosthesis with a BCI. Kapeller et al. (2014) classified three hand gestures: “open,” “peace,” and “fist.” In their decoding method, the presence of hand movement was classified first with a two-class LDA classifier (with an accuracy of 86.6 and 97.7% in their first and second subjects, respectively), and then a multi-class LDA detected the gesture (with an accuracy of 93.8 and 98.8%).

Furthermore, hand-gesture tasks have been used to investigate the ways the number of implanted electrodes could be reduced and confined to a smaller cortical area. Bleichner et al. examined two subjects with high-density ECoG grids implanted over a small area (2.5–5.2 cm^2^) in the hand-representing area. Four hand gestures corresponded to letters D, F, V, and Y of the American sign language (ASL) (Bleichner et al., 2016). Gesture classification was performed using a pattern-matching classification algorithm that was applied to ECoG spectral bands and local motor potentials (LMPs). An accuracy of 97 and 74% was reached for their first and second subjects, respectively. It was found that a selected electrode subset (two thirds of the total) was sufficient to reach the same classification accuracy as the accuracy achieved with all electrodes. In the study of Li et al. (2017), participants produced three hand gestures (“scissor,” “rock,” and “paper”). Classification accuracy with SVM classifier applied to spectral features was in the range 69.7–85.7% when performed offline and 80–82% during the online control of a prosthetic hand. The number of channels was reduced with a greedy algorithm. It was found that a subset of electrodes confined to a small cortical area was sufficient to maintain good decoding performance.

Xie et al. (2015) decoded different finger and hand movements from ECoG signals recorded intraoperatively in the motor cortex of awake subjects. They used an LDA classifier applied to the features extracted with an autoregressive model, and a waveform length feature that represented signal complexity. The intraoperative decoding accuracy (91.8 and 93.0% in two subjects) was comparable to the accuracy reached with the ECoG grids implanted for seizure monitoring (90.2 and 96.0% in the other two subjects). These results suggest that implementing BCI tasks during the implantation surgery could be useful for the adjustment of ECoG grid placement.

For proper reproduction of movements, movement onset needs to be decoded from neural activity in addition to the decoding of movement type. Early detection of the intention to move is important for BCI applications because it allows decreasing the delay between the brain activity and the response of the prosthetic device (Lebedev et al., 2008; Yanagisawa et al., 2011). Classification algorithms, such as LDA (Kapeller et al., 2014; Hotson et al., 2016) and SVM (Yanagisawa et al., 2011) have been used to detect movement onset based on ECoG recordings.

In conclusion, the classification and reproduction approach is suitable for neuroprosthetic applications where a finite set of motor outputs is sufficient, such as BCIs that enable sign language-like communications (Bleichner et al., 2016; Branco et al., 2017). Studies have shown that restoration of a finite set of movements is a practical BCI solution for amputees (Bruurmijn et al., 2017), and patients with hand paralysis (Shoham et al., 2001; Yanagisawa et al., 2012). Such BCIs could implement a shared control principle, where a general motor command is extracted from brain activity whereas the fine details of movements are handled by the controller of a prosthetic limb (Li et al., 2014).

### 3.3. Decoding of Motor Parameters as Continuous Variables

The third class of ECoG-based BCIs treats the parameters of movements, such as limb position and velocity, as continuous variables that are decoded from brain activity. Many studies have employed a center-out task for continuous decoding. During this task, subjects repeatedly perform cued or self-paced arm or hand movements from a center into different directions. These movements are usually converted into 2D or 3D movements of a cursor. The center-out task gained popularity after the studies of Georgopoulos et al. (1982) of the directional tuning properties of monkey motor cortical neurons. In ECoG studies with this design, four (Leuthardt et al., 2004; Reddy et al., 2009), six (Toro et al., 1994a), and eight (Leuthardt et al., 2004; Sanchez et al., 2008; Ball et al., 2009; Anderson et al., 2012; Wang et al., 2012; Nurse et al., 2015; Gunduz et al., 2016) targets locations have been used, all equidistant from the center. Center-out movements can be performed with a joystick (Reddy et al., 2009; Anderson et al., 2012; Wang et al., 2012), computer mouse (Kellis et al., 2012), stylus (Nurse et al., 2015), or the index finger moving on the surface of a touchscreen (Sanchez et al., 2008).

In a pioneering study that combined a center-out task with ECoG recordings in humans, Toro et al. (Toro et al., 1994a) evaluated tuning of ECoG in the 8–12 Hz band to the direction of arm movements. ECoG was sampled from the sensorimotor cortex and adjacent regions. Ten years later Leuthardt et al. (2004) analyzed a wider (0–200 Hz) range of frequencies and discovered directional tuning for various ECoG spectral bands. The center-out task was performed with a hand-held joystick and incorporated four or eight targets. Ball et al. (2009) decoded movement direction from ECoG during the execution of a center-out task and assessed the representation of directional information in different cortical areas. Their subjects performed self-paced center-out movements with their arms to four target locations. Decoding was performed with regularized linear discriminant analysis (RLDA) applied to either smoothed ECoG signals or different frequency bands. Decoding accuracy of 75% was achieved using the features calculated over the movement-execution period whereas 45% accuracy was achieved using the pre-movement period. ECoG channels corresponding to the hand and arm representing areas of the primary motor cortex were the most informative for the decoding. The analysis of additional data from a subject performing an eight-target task showed that ECoG activity (in the low-frequency and high-gamma bands) was cosine-tuned to the direction of arm movements. Anderson et al. (2012) investigated ECoG tuning to movement speed and velocity for center-out and tracing tasks performed with a force feedback joystick. ECoG recordings were conducted in multiple cortical areas. The strongest modulations to direction, speed, and velocity were observed in the primary motor cortex.

Wang et al. (2012) decoded movement direction with a time-varying dynamic Bayesian network. Center-out movements were performed with a joystick toward eight targets. Accuracy was quantified as the mean angular error between the actual and decoded direction; it was <90° in all subjects. Gunduz et al. (2016) reported a similar experimental design with center-out movements performed with a joystick, and eight targets. The task incorporated a delay period when the subjects prepared to move while holding the joystick still, which allowed decoding a person's planned direction of movement. Direction was decoded with a stepwise multilinear regression applied to high gamma activity and/or LMPs. The median angular error was in the range 62–70° across subjects. The authors observed directionally specific modulations of both high-gamma ECoG and LMPs during the delay and movement periods. Directionally tuned high-gamma activity was most prominent in the sensorimotor cortex whereas LMP modulations occurred in prefrontal cortices. The authors concluded that sampling directionally tuned ECoG from multiple cortical areas could improve the decoding of both planned and executed movements.

Reddy et al. (2009) enriched the center-out task with a tapping condition, which allowed testing how well center-out movements could be distinguished from the other types of movements. Center-out movements were performed with a joystick in response to arrows pointing in four possible directions. Additionally, subjects responded to a trigger cue (a square shown on the screen) by clicking on top of the joystick with the index finger. Decoding was performed using Naïve Bayes classifier applied to time-frequency features. Decoding accuracy for movement direction was in the range 83–96% for the preparatory period and 58–86% for the movement period. The trigger condition was distinguished with 72–93% accuracy from the center-out conditions.

Bundy et al. (2016) added the third dimension to the center-out task. Their subjects performed arm reaching movements with the starting position located at the center of a cube and cube vertices serving as targets. The kinematic parameters of movements were decoded with a hierarchical partial-least squares regression model. Correlation coefficients between the true and predicted kinematic parameters ranged 0.31–0.80 across subjects for speed, 0.27–0.54 for velocity and 0.22–0.57 for position. The final position was reconstructed with an accuracy of 49.0–66.2%.

Several studies employed reaching tasks that differed from the classical center-out paradigm. In the study of Kellis et al. (2012), patients moved a cursor with a computer mouse from an initial position at the bottom of the screen to the upper right or upper left corner; trajectories were decoded from ECoG with a Kalman filter. Sanchez et al. (2008) continuously decoded kinematic parameters in two tasks: a center-out task where subjects tracked smoothly varying trajectories extending from the center to predefined locations, and a target selection task where subjects performed reaches toward color-coded targets placed along the top edge of the screen. Cursor movements were decoded from ECoG frequency bands with a Wiener filter. Pistohl et al. (2008) had subjects acquire targets randomly positioned on a plane; hand coordinates were decoded with a Kalman filter. Schalk et al. (2007) reported highly accurate decoding of position and velocity using linear models for the task, performed with a joystick, where subjects pursued a target that moved counterclockwise along a circular trajectory. ECoG activity was cosine tuned to target angle, and decoding accuracy was comparable to the accuracy reported for monkeys implanted with MEAs.

In several studies, kinematics of finger movements was decoded from ECoG. Kubanek et al. (2009) extracted the time-course of finger movements from motor cortical activity. The subjects repeatedly flexed individual fingers in response to a visual cue. Decoding was performed with PaceRegression algorithm. Several other decoding algorithms of different complexity have been used for reproducing finger movements from ECoG, including switching linear model (Flamary and Rakotomamonjy, 2012; Liang and Bougrain, 2012) empirical mode decomposition (Hazrati and Hofmann, 2012), logistic-weighted regression (Chen et al., 2014), and LSTM (Du et al., 2018; Xie et al., 2018).

In addition to the aforementioned reaching tasks and finger-movement tasks, more complex motor tasks have been used in ECoG-BCI studies. Hammer et al. (2013) employed a game-like continuous one-dimensional motor task where subjects controlled the horizontal position of a car with a steering wheel. Position, velocity and acceleration were decoded with a linear regression algorithm. In the study of Nakanishi et al. (2013), participants repositioned blocks on a board. ECoG features were transformed into a three-dimensional arm trajectory with a sparse linear regression algorithm. In the subsequent study by the same group, subjects repositioned blocks with three different masses (Nakanishi et al., 2017). With this design, representations of intrinsic (e.g., muscle force) and extrinsic (e.g., target location) parameters of movements could be compared. ECoG recorded in the primary motor cortex was correlated mostly with the intrinsic parameters whereas ECoG recorded in pre-motor cortex was correlated with the extrinsic parameters. Wang et al. (2014) varied movement duration to investigate whether the entire movement course could be decoded from ECoG or only the values of motor parameters at movement onset. Wu et al. (2016) implemented a three-dimensional isometric force task where subjects exerted force in different directions without moving their arms. Directional information was extracted from the fronto-parietal ECoG recorded during both preparation and execution of the isometric task. The decoding algorithm incorporated a jPCA reduced-rank hidden Markov model (jPCA-RR-HMM), regularized shrunken centroid discriminant analysis, and LASSO regression.

Continuous-decoding BCIs based on ECoG recordings hold promise of eventually satisfying the requirements of paralyzed patients who need high-performance neuroprosthetic devices for restoration of mobility of their limbs. With a continuous-decoding neural prosthesis, patients would be able to execute a variety of movements in a near-normal way, where limb kinematics is constantly under the user's voluntary control and fine modifications of motor parameters could be done. Although a BCI with such an ideal control has not been demonstrated yet, recent advances in building fully implantable ECoG systems that perform continuous decoding (Vansteensel et al., 2016; Benabid et al., 2019) suggest that patients could improve in such BCI control through long-term practice that engages cortical plasticity mechanisms.

## 4. Decoding Algorithms

In this section, we describe in more detail the decoding algorithms used in ECoG-based BCIs. These algorithms bear similarity to the decoders for EEG-based interfaces, which have been covered in several review articles (Lotte et al., 2007, 2018; McFarland and Wolpaw, 2017). Here we review only the literature on the decoding of movements from ECoG.

ECoG recordings capture electrical potentials of large neuronal populations formed by synchronous dendritic potentials and spikes (Buzsáki et al., 2012). Decoding of motor parameters from ECoG is possible because modulations of neuronal population activity are consistently correlated with task events and changes in motor parameters (Anderson et al., 2012; Lebedev, 2014). Multichannel ECoG data contains spatial (i.e., where in the cortex) and temporal (i.e., when and how) information that could be used for decoding of movement characteristics. Spatial ECoG components reflect, according to the somatotopic cortical map of the body, the body part engaged in a motor action. Temporal ECoG components reflect the time-dependent changes of motor parameters, such as limb position, speed, and acceleration.

**Figure 2:**
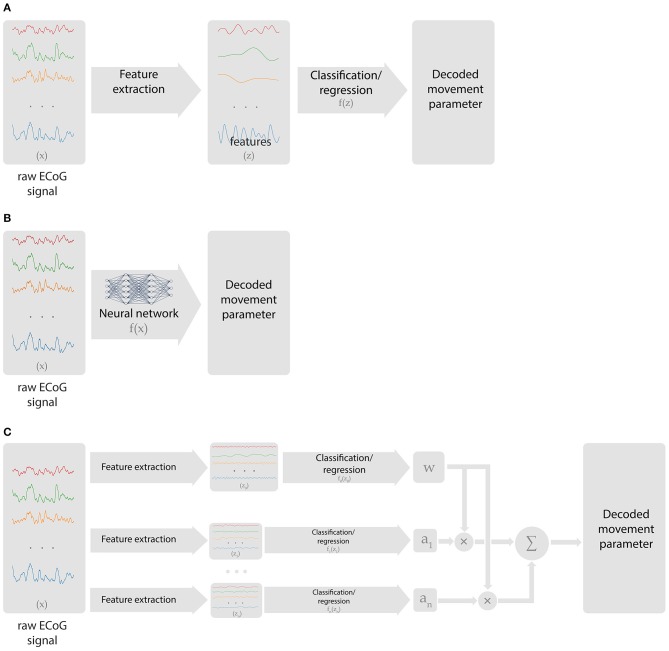
Types of data processing chains employed in ECoG-based BCIs. **(A)** Classical approach, where preselected features are extracted from ECoG recordings, followed by a classification or regression algorithm that generates BCI output. **(B)** Deep learning approach that handles both feature selection and decoding. **(C)** Hierarchical scheme with multiple decoders and processing chains that perform switching or relative weights adjustment.

An ECoG decoder takes multichannel ECoG data as the input and returns the signals of interest (the presence of movement, movement type, body part being moved, kinematic parameters, etc.) as the output. Many machine learning methods are applicable to this problem. The signal processing chain of a neural decoding algorithm comprises several blocks (Figure 2A). First, the raw data is transformed into features that contain information relevant to the BCI tasks. Ideally, these features should not contain redundant information. Next, a learning algorithm forms a decision rule that solves either a classification or regression problem. Classification algorithms solve the problem of matching an input with one of the predefined discrete classes. Regression algorithms match the input signals to the output continuously. For example, identification of the finger being moved is a classification problem, whereas decoding finger trajectory is a regression problem.

To properly set the decoder parameters, training data are needed that contain a sufficient number of examples of the inputs and their corresponding outputs. Based on the training data, a function is formed that, given the inputs from the dataset, returns values that are close to the corresponding desired outputs. A practicable decoder should be able to generalize to new data, that is, it should remain accurate when applied to the inputs not included in the training dataset. The case where decoding performs well for the training data but fails to work for the new data is called overfitting (Babyak, 2004). Overfitting often occurs when the decoder uses too many adjustable parameters, such as weights of the multiple linear regression. The presence of overfitting indicates that the transfer function is narrowly tuned to the anecdotal correlations between the input and output values taken from the training data rather than implements a generic transfer rule that reflects consistent input-output relationships. To avoid overfitting, feature space dimension reduction and appropriate regularization techniques should be employed. Thus, if an iterative approach is used to optimize decoder parameters, a proper stopping rule should be used to avoid overfitting.

Decoding algorithms have been developed that maintain generalization even when the sampled neural signals drift over time. Thus, Paul et al. (2017) used the higher-order statistics of ECoG bispectrum to overcome the difficulties decoding signals that were recorded during multiple task sessions. Their algorithm extracted signal features that were retained after a session-to-session transfer. This finding is consistent with the results of previous EEG-based studies (Shahid and Prasad, 2011; Das et al., 2016).

An additional important requirement is the versatility of training data, which means that the space of movements should be covered during sampling in such a way that the decoder interpolates to new data points rather than extrapolating to them. Practically, this means that experimental settings used to collect training data should be similar to the settings for online BCI control, including both the characteristics of movements and neural activity patterns. In the case of a mismatch between the training and online-BCI conditions, adjustments of the decoder may be needed to improve BCI performance.

### 4.1. Spectral Features

An important advantage of ECoG recordings compared to EEG is the wider range of signal frequencies that contain information useful for BCI control. ECoG activity comprises multiple frequency bands, from the low frequency activity (below 1 Hz) to high gamma (50–400 Hz). Some of these spectral components are clearly rhythmic, with clear peaks present in ECoG spectra (Miller et al., 2007). Each frequency band has specific functional correlates, which allows implementing decoders that capture different aspects of the behavioral tasks, such as responses to stimuli, transition from rest to movement, characteristics of limb kinematics, and engaging different body parts. An ECoG decoder that uses multiple frequency bands simultaneously is potentially more accurate and versatile compared to the decoder based on a single spectral band.

To extract task-related spectral features, ECoG signal is either bandpass filtered (Liang and Bougrain, 2012; Chestek et al., 2013; Nakanishi et al., 2013) or converted into the frequency domain using non-parametric methods, such as Fourier transform (Chin et al., 2007; Miller et al., 2007; Blakely et al., 2009; Reddy et al., 2009; Ryun et al., 2014), multitaper methods (Ball et al., 2009; Kellis et al., 2012; Pistohl et al., 2012; Elgharabawy and Wahed, 2016), parametric techniques, such as autoregressive model estimation (Leuthardt et al., 2004; Schalk et al., 2007; Kubanek et al., 2009; Wang et al., 2012; Xie et al., 2015), and the maximum entropy approach (van Vugt et al., 2007; Collinger et al., 2014; Bundy et al., 2016; Gunduz et al., 2016). Spectral features can be also extracted with filter bank methods, such as Gabor filters (Liu et al., 2010; Elghrabawy and Wahed, 2012; Elgharabawy and Wahed, 2016; Wu et al., 2016). Ideally, neural signals should be processed in such a way that an optimal trade-off is reached between the temporal and spectral resolution.

**Figure 3:**
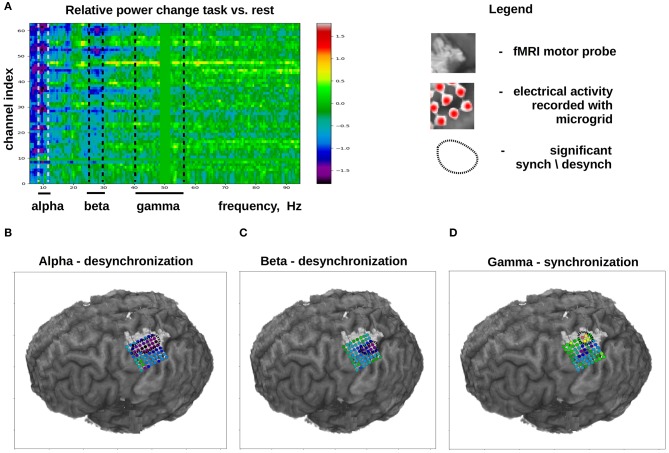
Typical changes in ECoG activity that occur during the execution of a motor task (in this case, finger flexion). Task-related activity is compared to ECoG activity recorded during a rest period. **(A)** Channel index × spectral frequency diagram with the color-coded values representing desynchronization index calculated as $2\frac{P_{task}-P_{rest}}{P_{task}+P_{rest}}$. **(B–D)** Cortical distribution of desynchronization index for different ECoG frequency bands. **(B)** Data for the alpha band. It can be seen that, during a motor task, alpha-band activity is desynchronized over a large portion of the sensorimotor cortex. **(C)** Data for the beta band. Beta band activity is desychronization over a more compact cortical area as compared to the alpha-band. **(D)** Data for the beta band for the high frequency gamma activity (40–60 Hz), which exhibits a pronounced synchronization over a small cortical area. The light-gray shaded spot shows the localization of the hand-related sensorimotor are obtained with fMRI.

ECoG mu (8–12 Hz) and beta (18–26 Hz) rhythms recorded in the sensorimotor are commonly used for decoding movements from ECoG. These oscillations are thought to reflect the activity in corticothalamic loops (Schalk and Leuthardt, 2011). The mu and beta rhythms are typically not confined to local cortical areas but rather occur over large surfaces (Brunner et al., 2009). Movement and motor imagery cause desynchronization (i.e., decrease in amplitude) of these rhythms, which allows implementing BCIs that detect movement onset and time course. While ECoG recordings are useful for measuring the mu and beta activity, approximately the same measurements, albeit with a lower spatial resolution, could be accomplished with EEG recordings, which are suitable for monitoring cortical rhythms below 40 Hz. By contrast, gamma-band activity (40 Hz and higher) cannot be reliably recorded with EEG due to signal contamination by facial EMG activity that belongs to the same frequency range. Yet, gamma activity is reliably sampled with ECoG. ECoG activity in the gamma band matches the activity of single neurons in the same area (Buzsáki et al., 2012) and, unlike the slower rhythms, it is not widespread but rather occurs in local cortical areas (Schalk and Leuthardt, 2011). These properties make ECoG gamma activity suitable for decoding based on cortical location and for decoding specific aspects of movement planning and execution with the accuracy comparable to the decoding from neuronal spikes (Anderson et al., 2012; Gunduz et al., 2016). ECoG gamma recordings are also useful to study cognitive mechanisms (Sturm et al., 2014). Thus, high-frequency ECoG components are especially valuable for implementing BCIs of different kinds. Figure 3 shows the typical changes that occur in different ECoG frequency bands during the execution of a motor task.

At the lower end of ECoG spectrum (<2 Hz), ECoG low-frequency component (LFC) has been shown to be applicable for BCI decoding because it contains information about movement timecourse and kinematics (Mehring et al., 2003; Rickert et al., 2005; Pistohl et al., 2008; Ball et al., 2009; Hammer et al., 2013). LFC can be extracted, for example, by smoothing the signal with Savitzky-Golay filters (Pistohl et al., 2008, 2012; Ball et al., 2009). Schalk et al. (2007) called this component local motor potential (LMP) and computed it as a running average. LMP is modulated during motor behaviors, so it can be used for decoding limb kinematics (Kubanek et al., 2009; Acharya et al., 2010; Fifer et al., 2012; Wang et al., 2012; Chen et al., 2014; Hotson et al., 2014; Bleichner et al., 2016; Bundy et al., 2016; Wu et al., 2016). Hammer et al. suggested that LFC phase is more informative for motor decoding than LFC magnitude (Hammer et al., 2013). While LFC is highly informative for decoding, it can be easily contaminated by mechanical and electrical artifacts caused by the movements of the limbs and recording equipment. Because of this issue, a special care should be taken to minimize the artifacts, remove them from the data programmatically and ensure that they are not utilized for decoding.

Besides spectral band power modulations, within-band and across-band coupling features appear to be informative on movement intentions. Thus, Brunner et al. (2005) found extra information in the phase coupling between different ECoG channels, measured as phase locking value (PLV). This method worked well when applied to the beta and gamma bands.

Several connectivity measures have been applied to the analysis of ECoG. Bayesian networks (TV-DBN) and eigenvector centrality analysis have been used to identify brain regions relevant to motor tasks (Newman et al., 2015). Benz et al. (2012) used TV-DBN to quantify task-related changes in connectivity and to decode hand kinematics. With this approach, higher accuracy was achieved compared to spectral feature decoders. Babiloni et al. (2017) utilized lagged linear connectivity (LLC) between several cortical areas in the delta-theta (<8 Hz) band to distinguish action execution from action observation.

### 4.2. Spatial Features

Decoder accuracy is known to improve with increasing number of recording channels (Nicolelis and Lebedev, 2009). In addition to the mere number of channels, improvements in decoding can be gained by accounting for the spatial arrangement of recording sensors, such as the arrangement of electrodes in an ECoG grid. The procedure that improves decoding using the information about the electrode locations is referred to as spatial filtering. Spatial filters treat different ECoG channels as coordinates for multivariate data sampling. This coordinate system is transformed by the filter to improve decoding. For example, spatial filtering could be used to reduce data dimensionality or improve separability of different observations.

The initial spatial filtering is usually accomplished with the reference schemes utilized during ECoG recordings. Common average reference (CAR) is typically used as a simple denoising technique (Schalk et al., 2007; Kubanek et al., 2009; Wang et al., 2012). This method reduces noise that is common to all recording channels but it does not handle channel-specific noise and it may also introduce noise into otherwise clean channels. Several alternative filtering techniques have been proposed to address these problems. Morales-Flores et al. (2014) developed a non-supervised algorithm where the spatial filter coefficients are adjusted using a steepest descent method that minimizes the variance on differences of the linear combination of ECoG channels. This approach improved the decoding of finger flexions from ECoG when compared to the data produced by CAR. Liu et al. (2015) considered the problem of the introduction of channel-specific noise when CAR is applied to the channel sets containing noisy channels. They tested several types of unsupervised spatial filters and techniques for detecting artifacts. After the noisy channels were automatically removed, data contamination was reduced. Moreover, they developed a median average reference filter that reduced channel-specific noise even when the noisy channels remained in the set.

Principal component analysis (PCA) is widely used in conjunction with spatial filtering, primarily for dimensionality reduction (Freeman et al., 2000; Boye et al., 2008). This method transforms the original data into principal components, which are uncorrelated with each other and are created in such a way that the first several components capture the largest possible amount of variance in the data. The principal components are quantitatively characterized in terms of how much variance (i.e., information contained in the data) they comprise. After the PCA transformation, the least informative (or least powerful) components can be discarded, reducing data dimensionality. This approach is, however, not optimal in the cases where information is present in the low-power features of the ECoG signal. In some cases, dimensionality reduction techniques, such as PCA, are applied not only to ECoG signals but also to motor parameters (Liu et al., 2010; Samiee et al., 2010; Hotson et al., 2014). This is particularly useful when movements are unconstrained. In this algorithm, the decoder first generates output in PCA coordinates, and this output is then converted into the original coordinates. Canonical correlation analysis is another technique that can handle high multidimensionality of both ECoG and movement data. This method performs a linear transformation that maximizes the correlation between the ECoG activity and movements (Spüler et al., 2016).

Common spatial patterns (CSP) is a spatial filtering technique that is often used in EEG- and ECoG-based BCIs to extract features that are useful for classification (Kapeller et al., 2014, 2015; Xie et al., 2015; Jiang et al., 2017). When two classes of observations are used, CSP maximizes the ratio of their variances to increase the separability of the two classes. After the CSP transformation, dimensionality reduction can be carried out based on the separability of the two classes in different dimensions. Additionally, CSP performs more robustly and exhibits better generalization properties when preceded by a separate dimension reduction step (Nicolae et al., 2017).

Source reconstruction methods are applicable to improve the performance of ECoG-based BCIs. The use of dynamical spatial features obtained from the reconstructed cortical current source density has been already shown to drastically improve the decoding accuracy in the MEG and EEG based BCIs where subjects generate outputs using motor imagery (Lin et al., 2013; Edelman et al., 2019). Raw ECoG recordings better reflect the surface distribution of cortical sources compared to non-invasive measurements (Schalk and Leuthardt, 2011). Yet, the activity of sources located deep in the sulci spreads into several recording channels and therefore can not be assessed selectively in the raw data. As a solution to this problem, a sufficiently fine model can be built that describes the relationship between the activity of neuronal sources and the ECoG measurements (Gramfort et al., 2010). Based on such forward model, reasonably accurate current source density reconstructions can be obtained for neuronal sources located within 1 cm from the cortical surface (Zhang et al., 2008; Pascarella et al., 2016; Todaro et al., 2018). We foresee that such reconstruction of sources from ECoG will be useful for BCI decoding by providing decoding algorithms with the inputs that discern the activity of more compact cortical areas compared raw ECoG data. To fully benefit from this approach, care needs to be taken to accurately determine grid location with respect to the cortical surface. In addition to geometric calculations, the techniques exploiting functional data-driven methods based on maximizing model evidence (Henson et al., 2009) could improve the performance of these methods.

In addition to the methods described above that perform spatial filtering and/or reduce data dimensionality (Gu et al., 2012), the decoding accuracy benefits from techniques to determine the most informative features for classification, such as requesting a certain separation in power for a certain ECoG spectral band for different movements (Ryun et al., 2014), choosing features strongly correlated with the task (Leuthardt et al., 2004), successively adding features correlated to the class and not correlated to the previously selected features (Schalk et al., 2007), or choosing features according to a scatter-matrix based separability (Samiee et al., 2010). Several filter selection algorithms utilize a wrapper-based approach, where features are scored using the learning algorithm that is then used for regression or classification (Gu et al., 2012). In this approach, the feature set is enhanced in consecutive steps, where features are added to the previous feature set to improve decoding accuracy estimated with cross-validation (Liang and Bougrain, 2012; Wang et al., 2012; Elgharabawy and Wahed, 2016; Li et al., 2017). When following these strategies, one should bear in mind that ECoG features assumed to be useful could be contaminated by noise that is accidentally correlated to the parameters being decoded.

### 4.3. Classification and Regression

Starting with the report of Levine et al. (1999) on movement-related ECoG patterns, pattern matching techniques have been applied to derive motor commands from ECoG recordings. Thus, movement-related ECoG desynchronization was detected using an average ECoG template and cross-correlating it with ECoG samples (Huggins et al., 1999). More complex features can be used for the same purpose (Graimann et al., 2003). Such pattern-matching approach has been successfully used to classify multiple movement types (Bleichner et al., 2016) and to implement BCI control (Levine et al., 2000).

As explained above, the capacity to generalize to new data is essential for both classification and regression algorithms. Since the number of features is often large, regularization methods are applied to prevent overfitting. Algorithms with fewer parameters are less susceptible to overfitting and often perform no worse than more complex algorithms (Marjaninejad et al., 2017).

For decoding ECoG into discrete classes, linear discriminant analysis (LDA) is often used (Ball et al., 2009; Samiee et al., 2010; Pistohl et al., 2012; Xie et al., 2015; Bleichner et al., 2016; Jiang et al., 2017). Classification can be performed as well using other algorithms, such as k-nearest neighbor method (Chin et al., 2007; Samiee et al., 2010; Paul et al., 2017) and Naïve Bayes classifier (Chestek et al., 2013).

Support vector machines (SVM) is another class of models that solve the problem of separating samples of different classes by maximizing the margin between them. This group of algorithms is versatile and allows constructing highly non-linear decision surfaces. Linear kernel is often used to prevent overfitting and ensure robustness (Yanagisawa et al., 2009, 2011; Ryun et al., 2014; Elgharabawy and Wahed, 2016). Additionally, radial basis functions can be employed (Wang et al., 2012). The disadvantage of this approach is that kernel selection remains largely heuristic and is usually performed via some sort of cross-validation that requires additional data.

For continuous decoding of motor parameters from ECoG, linear models are often used, including linear regression (Schalk et al., 2007; Liang and Bougrain, 2012; Hammer et al., 2013; Hotson et al., 2014; Gunduz et al., 2016) and its modifications designed to reduce overfitting (Kubanek et al., 2009; Nakanishi et al., 2013). Sanchez et al. used the Wiener filter, a linear model, to decode movement trajectory (Sanchez et al., 2008). Pistohl et al. (2008) and Kellis et al. (2012) utilized the Kalman filter, which better handles non-stationary inputs. Wang et al. (2012) employed a modification of dynamic Bayesian network to capture non-stationarity in the temporal and spatial ECoG characteristics.

Several studies utilized prior knowledge of the task performance to improve decoding. Schalk and Leuthardt (2011) developed a Bayesian decoding model that incorporated constraints on finger flexion. Wu et al. (2016) employed a hidden Markov model that highlighted rhythmic task behavior. Saa et al. (2016) enhanced their decoding algorithms with the assumption that subjects do not perform rapid changes between movement and rest.

Hierarchical algorithms (i.e., the ones that stack several models) are often used to enable online BCI tasks. In these schemes, different regression and classification tasks are performed in a certain order (Figure 2C). Yanagisawa et al. (2011) and Hotson et al. (2016) used a hierarchical algorithm, where one model classified between rest and movement and detected movement onset and the second model classifies movement type. In several studies, switching between regression models was performed based on a classification algorithm (Flamary and Rakotomamonjy, 2012; Bundy et al., 2016; Elgharabawy and Wahed, 2016). Additionally, Chen et al. developed an algorithm where the output of one model was used to weigh the output of the other model to improve prediction accuracy (Chen et al., 2014).

Artificial neural networks are the class of algorithms that handle complex, non-stationary patterns of brain activity. They can be applied to both classification and regression problems. The primary advantage of artificial neural networks is their versatility. With sufficient number of model parameters (units or neurons), complex neural patterns can be processed. While shallow neural networks with few layers are useful for decoding, during the last several years deep neural networks containing many layers have significantly advanced. Advantages of deep learning models include their ability to automatically extract features useful for decoding rather than hand-engineering them (Figure 2B) and to generate representations at multiple levels of abstraction.

Deep learning is rapidly gaining popularity as a BCI decoding method. In the last few years, deep learning algorithms have been applied to ECoG data processing (Roy et al., 2019), seizure forecasting (Meisel and Bailey, 2019), language mapping (RaviPrakash et al., 2018), and speech decoding (Livezey et al., 2018; Angrick et al., 2019a,b). Several studies have already employed deep learning for decoding movements from ECoG. Xie et al. (2018) decoded finger trajectory with high accuracy using LSTM recurrent neural network. Du et al. (2018) applied LSTM to the same data and implemented real-time control of a robotic arm. Wang et al. (2018) employed a deep model to detect the upper body joints movement based on both ECoG recordings and video data. Pan et al. used recurrent neural networks that recognized temporal dependencies in ECoG signal for rapid and robust gesture decoding (Pan et al., 2018). We foresee further and fruitful development of deep learning approaches for ECoG-based BCIs. This is because of several advantages of this approach. Deep learning architectures applied to electrophysiological data (Roy et al., 2019) perform on par or slightly better than the classical algorithms and do not require neural features to be defined upfront. While such automated processing can be considered as an advantage, BCI researchers still would want to better understand the processing chain performed by a deep learning algorithm, and ideally to relate the processing steps to certain physiological characteristics of the recorded neural signals. Such understanding of the representation of information deep architectures employed for decoding purposes is crucial in order to assess validity of the obtained solutions (Hammer et al., 2013). Thus, it is important to understand the contribution to decoding from different types of neuronal activity, such high-frequency ECoG components better corresponding to neuronal discharges and low-frequency ECoG reflecting synchronization of large neuronal populations (Aoki et al., 1999; Chestek et al., 2013). Additionally, one needs to be able to distinguish causal decoding that captures commands generated by the brain from the decoding based on the peripheral reafferent signals resulting from overt behaviors (Livezey et al., 2019). With a better understanding of these functional relationships, BCI developers can make full use of the information carried by the neural signals, avoid inadvertent uses of informational confounds, establish practical utility of their algorithmic solutions, and gain fundamental neurophysiological insights.

## 5. Software

ECoG-based BCIs can be implemented using several currently available software packages that perform real-time processing of multichannel neural data. OpenVIBE (Renard et al., 2010) is one popular project that offers tools for visual programming and scripting signal processing pipelines. Experimental task descriptions are saved as XML files. OpenVIBE is closed source software. Another popular closed source package for implementing BCIs is BCI2000 (Schalk et al., 2004). BCI2000 is written in C/C++. It incorporates several algorithms for processing multichannel recordings. In our laboratory, we have recently developed NFBLab^1^, an open-source software written in Python for implementing a variety of BCI designs (Smetanin et al., 2018b). This software accepts ECoG signals as inputs, as well as EEG and MEG recordings and synchronizes them with motion-tracking information and other multimodal data. Lab Streaming Layer^2^ protocol is used to interface NFBLab to data acquisition devices. NFBLab implements temporal and spatial filters for selecting signal feature and removing artifacts. Inverse solvers that generate source-space representation of multichannel inputs are implemented via an interface to MNE-Python software (Gramfort et al., 2014). Additionally, NFBLab incorporates algorithms that reduce processing latency (Smetanin et al., 2018a).

Several standard general purpose libraries are available for implementing deep learning approaches, such as *PyTorch, TensorFlow*, and *Keras*. Currently, only a few wrappers are available implementing specific functions that facilitate electrophysiological data processing. The *Braindecode* toolbox by Schirrmeister et al. (2017) is based on *PyTorch* and supports trial-wise and cropped decoding of raw EEG data. This toolbox is applicable to ECoG data. A novel software package *MNEFlow* for dealing with EEG/MEG data is currently being developed^3^ with three architectures implemented so far: *LFCNN, VARCNN* (Zubarev et al., 2018), and *EEGNet*. The latter architecture (Lawhern et al., 2018) implements a compact convolutional network; it is available for download^4^. While these libraries have not been developed to specifically process ECoG, they can be rapidly adapted to process ECoG data.

The developers of decoding algorithms can utilize open ECoG datasets containing data from movement and motor imagery tasks. For instance, dataset 4 from international BCI competition IV^5^ contains data for finger movements. This dataset is often used as a benchmark for BCI decoders that classify the finger being moved and/or perform continuous reconstruction of finger movements. The other ECoG dataset from BCI competition III^6^ contains recordings from several experimental sessions, so it is useful for testing how well a BCI decoder generalizes from one session to another. Researches from Brunton Lab made available a large annotated dataset^7^ containing long-term ECoG recording along with joint kinematics. Stanford Collection of ECoG Data^8^ includes recordings from 250 subjects conducted over an 8-years period. This dataset includes ECoG recordings from the sensorimotor cortex in patients performing motor tasks.

## 6. Discussion

Over the last two decades we observe a growing number of ECoG-based BCI studies in patients who underwent implantation for clinical purposes. This research is clinically relevant and holds promise to provide new treatments for people suffering from severe motor and sensory disabilities caused by such conditions as spinal cord injury, stroke and amyotrophic lateral sclerosis. At the same time, these studies have already provided benefits to the patients and materialized in take-home BCI systems for text-dialing purposes (Brunner et al., 2011), novel safer solutions for passive speech mapping of eloquent cortex during neurosurgery (Taplin et al., 2016; Sinkin et al., 2019) and wireless ECoG devices (Matsushita et al., 2018) that reduce septic risks and can be employed for chronic monitoring of patients with epilepsy. In recent years, it has become clear that ECoG-based BCIs are a viable approach to restoration and rehabilitation of motor functions. ECoG recordings are useful for decoding such motor parameters as movement onset (Wang et al., 2012; Pistohl et al., 2013), movement type (Pistohl et al., 2012; Ryun et al., 2014), and limb trajectory (Pistohl et al., 2008; Nakanishi et al., 2013; Eliseyev and Aksenova, 2014; Xie et al., 2018). These decoded signals can be then sent to external devices, such as hand prosthesis with many degrees of freedom (Yanagisawa et al., 2011; Hotson et al., 2016) or a lower-limb exoskeleton (Vansteensel et al., 2016; Benabid et al., 2019). ECoG-based BCIs can control two-dimensional and three-dimensional movements of a cursor or a prosthetic arm (Anderson et al., 2012; Yanagisawa et al., 2012). Several kinematic parameters can be extracted from ECoG, including position, velocity, and acceleration (Hammer et al., 2013). Extrinsic variables, such as target location, can also be also decoded from ECoG and utilized for BCI control (Nakanishi et al., 2017). The recently developed fully implantable ECoG-based BCIs (Vansteensel et al., 2016; Benabid et al., 2019) have extended the functionality of such systems as they enable long-term operations and engage cortical plasticity. With the rapid development of new technologies for high-fidelity ECoG recordings (Viventi et al., 2011; Akinwande et al., 2014; Khodagholy et al., 2015) and of neural decoding methods (Faust et al., 2018; Richards et al., 2019), ECoG-based BCIs will likely continue to improve.

ECoG-based BCIs are clinically relevant due to their safety as compared to the intracortical implants (e.g., Utah array) and have a better spatial and temporal resolution than non-invasive, EEG-based BCIs. At the same time, the ECoG grids cover relatively cortical areas which allows to take advantage of the spatial-temporal encoding principles implemented by the brain. Such large-scale recordings improve BCI accuracy by allowing for simultaneous access to the information processed by many brain regions involved in programming and execution of movements.

Broad spectral and spatial extent of ECoG recordings open the opportunity to explore at various scales interregional interactions between and within several frequency bands from delta-band (Gunduz et al., 2016) correlates of movement, desynchronization in the alpha and beta bands in spatially distributed task-relevant cortical areas to more localized synchronization in the high gamma range and cross-frequency coupling between bands and specific cytoarchitectonic assemblies. This flexibility leads to significant variability in the choice of features, decoded parameters and decoding models witnessed in the range of described ECoG studies. Thus, depending on the clinical needs, different ECoG components and associated neurophysiological phenomena can be utilized in practical BCI system.

In recent years, an active development of the decoding algorithms is underway. Several strategies have been particularly useful, including switching models, adapting algorithms, and the decoders utilizing prior information on movement characteristics and the nature of physiological processes. Even more versatile methods are currently being developed, such as those based on deep learning which allows for capturing complex relationship between motor parameters and ECoG characteristics.

We foresee that the next series of major advances will be made in bidirectional BCI technology that combines motor-control loops with sensory feedback provided by cortical stimulation and/or sensory substitution methods (Wilson et al., 2012; Cronin et al., 2016; Hiremath et al., 2017). The development of bidirectional ECoG-based BCIs will bring new challenges for modeling the complex relationships between ECoG signals and different motor and sensory parameters. Previous studies have reported a range of promising results regarding the possibility of building BCIs that employ ECoG recordings to enable motor functions. With the rapid developments in ECoG technologies (Shokoueinejad et al., 2019), surgical implantation procedures and mathematical algorithms for neural decoding, it is reasonable to expect that a variety of practical, fully-implantable (Vansteensel et al., 2016) ECoG-based neural prostheses will emerge for enabling motor and sensory functions to neurologically impaired patients.

## Author Contributions

KV wrote the first draft of the manuscript. KV, ML, AK, and AO revised the manuscript. All authors contributed to the final revision, read, and approved the submitted version.

### Conflict of Interest

The authors declare that the research was conducted in the absence of any commercial or financial relationships that could be construed as a potential conflict of interest.

## Footnotes

^1^https://github.com/nikolaims/nfb/wiki/Experiment-file-structure

^2^https://github.com/sccn/labstreaminglayer

^3^https://mneflow.readthedocs.io

^4^https://github.com/vlawhern/arl-eegmodels

^5^http://www.bbci.de/competition/iv/

^6^http://www.bbci.de/competition/iii/

^7^https://www.bingbrunton.com/data

^8^https://purl.stanford.edu/zk881ps0522
